# Endoscopic Ultrasound Guided Embolization of a Pancreatic Pseudoaneurysm

**DOI:** 10.4021/gr500w

**Published:** 2012-11-20

**Authors:** Paul M. Robb, Paul Yeaton, Thomas Bishop, John Wessinger

**Affiliations:** a Virginia Tech-Carilion School of Medicine, Department of Internal Medicine, Roanoke, USA; b Virginia Tech-Carilion School of Medicine, Department of Gastroenterology, Roanoke, USA; c Virginia Tech-Carilion School of Medicine, Department of Interventional Radiology, Roanoke, USA

**Keywords:** Chronic pancreatitis, Pseudoaneurysm, Endoscopic ultrasound, Interventional radiology, Embolization

## Abstract

Pseudoaneurysms are rare complications of chronic pancreatitis and are associated with a high mortality. In this article we demonstrate a novel utilization of endoscopic ultrasound (EUS) technology to embolize a large pancreatic pseudoaneurysm when gold standard therapies had proven futile.

## Introduction

Pancreatic pseudocysts complicate pancreatitis in approximately 40% of cases [1-3]. Proposed pseudocyst pathogenesis includes development as a consequence of acute inflammation or the obstruction of the pancreatic duct (protein plug, calculus, or fibrosis) [1-3]. CT is a useful initial imaging modality as it defines the presence of calcifications and is rapidly performed and widely available [1, 2, 4]. The standard of care for pseudocysts is often supportive, though when indicated, Endoscopic Ultrasound (EUS) has become a common technique to intervene in their management.

Pancreatic pseudoaneurysms have an incidence of less than 10% and are a potentially life-threatening complication of inflammatory pancreatitis, typically complicating pseudocysts. Non-enteral bleeding in the setting of pseudocysts requires swift evaluation and treatment. Pseudoaneurysms are commonly asymptomatic and likely to be detected coincidentally with CT. Presumably enzymatic autodigestion leads to vascular wall injury, with subsequent ballooning of the vascular wall. Hemorrhage is associated with a mortality rate as high as 40% [1, 3]. Treatment of pseudoaneurysms includes coil embolization, covered stent placement, thrombin injection, and open surgical repair, but most commonly endovascular coil embolization is utilized [2].

We report a patient with complicated pancreatitis in whom endovascular therapy of a pseudoaneurysm proved impossible, who was successfully treated with endoscopic ultrasound directed embolization of an arterial pseudoaneurysm.

## Case Report

**Figure 1:**
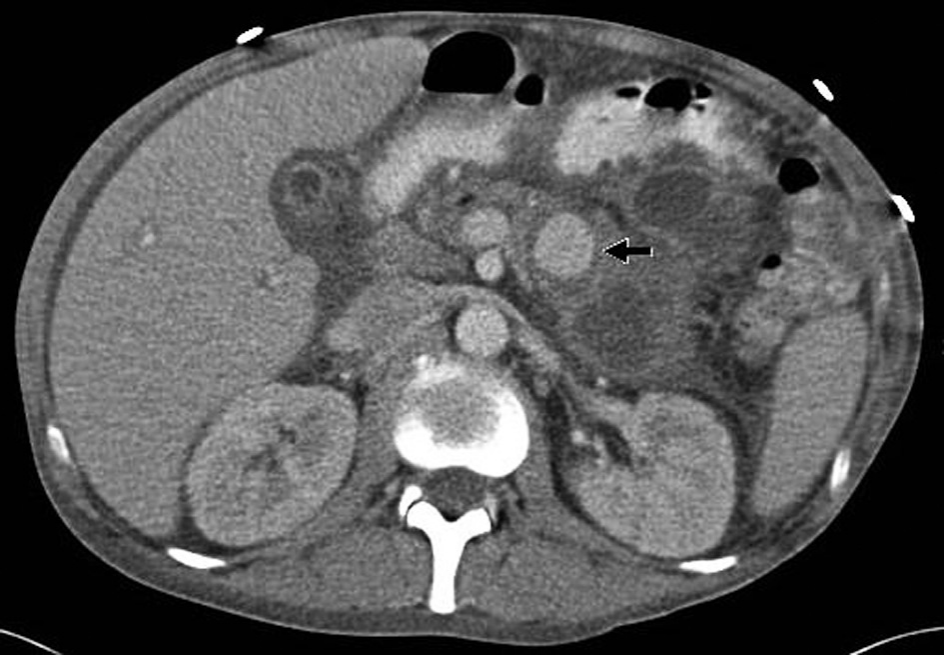
Pre-procedure CTA demonstrating diffuse pancreatitis with pseudocyst and a large pseudoaneurysm arising from a branch of the superior mesenteric artery.

A 54-year-old male with chronic calcific pancreatitis presented with an infected pseudocyst. Abdominal CT with contrast demonstrated complex collections in the pancreatic head and left upper quadrant, with a 3 cm pseudoaneurysm arising from a small branch of the superior mesenteric artery (Fig. 1). Endoscopic ultrasound-guided pseudocyst drainage was performed, noting the proximity and access of the pseudoaneurysm from the gastric lumen. Due to irregular arterial anatomy, pseudoaneurysm embolization was attempted unsuccessfully three times.

**Figure 2:**
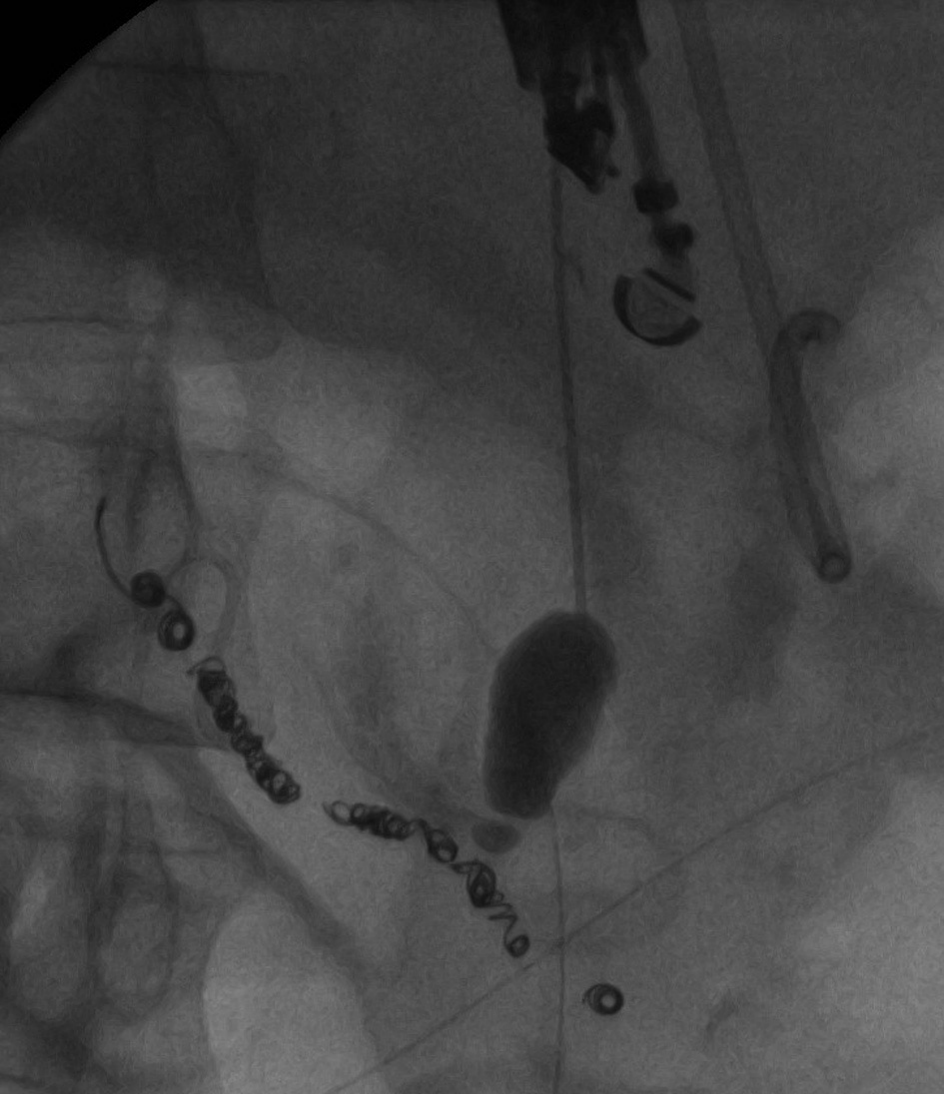
Opacification of the arterial pseudoaneurysm with a 19-gauge Cook needle, with retrograde filling of the feeding vessel.

Following the third attempt, a combined procedure was performed, integrating both interventional radiology and endoscopic ultrasound services. A GF-UC140P curved linear array echoendoscope (Olympus America, Center Valley, USA) was advanced into the gastric lumen identifying the pseudoaneurysm. Doppler was used to identify the relevant vascular anatomy. A 19-gauge Cook EchoTip needle (Cook Medical, Bloomington, Indiana) was used. The stylet was removed and the needle flushed with sterile normal saline. The needle was advanced through the gastric lumen into the pseudoaneurysm. Location was confirmed by aspiration of blood (Fig. 2).

**Figure 3:**
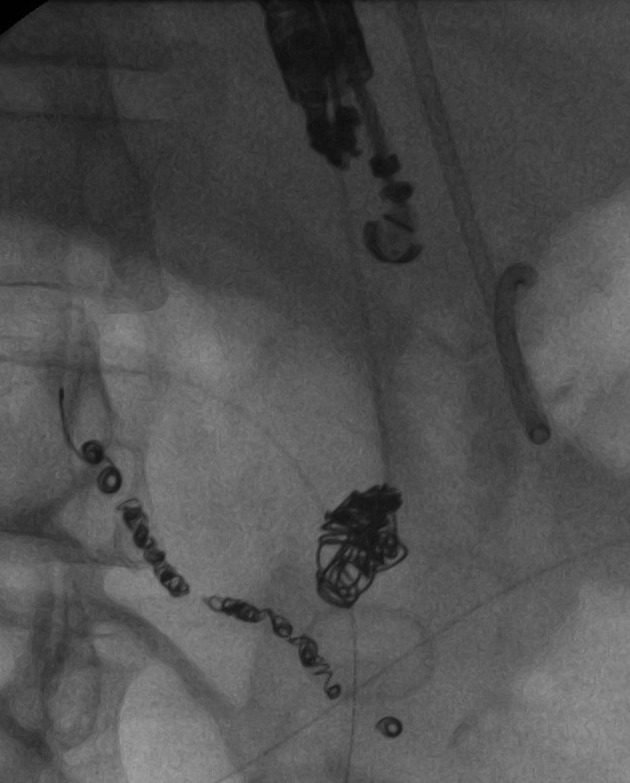
Impaction of Nester Embolization Coils into the arterial pseudoaneurysm through the 19-gauge needle.

**Figure 4:**
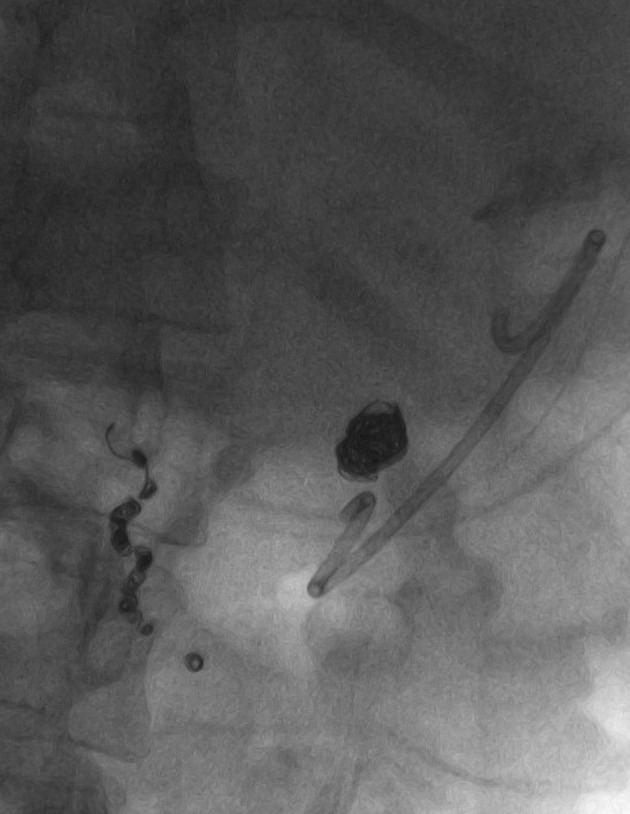
Final image following impaction of the Nester Embolization Coils.

**Figure 5:**
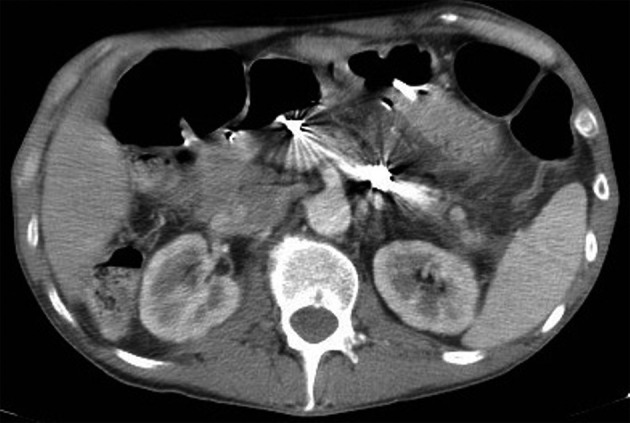
CT scan performed 5 months following EUS guided arterial pseudoaneurysm embolization.

Pseudoaneurysm embolization was performed under fluoroscopic visualization through the 19-gauge needle utilizing multiple 0.035” Nester Embolization Coils (Cook Medical, Bloomington, Indiana) of varying sizes (Fig. 3, 4). Contrast injection through the needle demonstrated the pseudoaneurysm to be adequately packed with minimal retrograde flow into the arterial feeding branch. The patient returned for follow-up five months following embolization with a contrast CT (Fig. 5). The pseudocyst and inflammation had resolved and the patient had returned to his premorbid function

## Discussion

Pseudoaneurysms are a serious complication of chronic pancreatitis and have an incidence of hemorrhage ranging from 1.4-8.4% [1, 3]. The most common vessels involved include the splenic (30-50%), gastroduodenal (17%), and pancreaticoduodenal arteries (11%) [3]. Ultrasound with color Doppler, CT and digital subtraction angiography enable proper pseudoaneurysm imaging for assessment and treatment [3-6]. Pseudoaneurysm rupture requires emergency angiography, which in recent history has reached a sensitivity of 100% and is regarded as the diagnostic modality of choice. Angiography demonstrates superior detection of both bleeding and non-bleeding varieties of pseudoaneurysms [1, 3].

This case emphasized the degree of difficulty involved in successful embolization of the pseudoaneurysm. Direct visualization under ultrasound guidance led to the success of the procedure. The US-guided transgastric introduction of the needle into the pseudoaneurysm implemented the Poiseuille equation of fluid dynamics. Intuitively, introducing a needle into a highly pulsatile arterial mass would result in hemorrhage. However, the physics of fluid dynamics dictates that a longer needle attenuates pressure change from the high flow arterial pseudoaneurysm to the stomach resulting in a reduced flow velocity.

Endoscopic ultrasound provides precise pseudoaneurysm location along with collateral vessel assessment, providing a safer intervention with reduced mortality and complications. Success rates of trans-catheter embolization range from 78-100% and have less than 3% peri-procedural mortality [1, 3]. The success of the procedure is not without the major limitation of having a relative blind approach, which carries significant risk of perforation and hemorrhage [5, 6]. Though endoscopic ultrasound-guided treatment is not the gold standard for pseudoaneurysms, this case demonstrates its success, which to our knowledge has not yet been reported. This case demonstrates that with the proper skill, this procedure can be successfully implemented with minimal complications once other accepted modalities have proven futile.
